# Complex excitability and “flipping" of granule cells: An experimental and computational study

**DOI:** 10.1371/journal.pone.0339418

**Published:** 2026-02-02

**Authors:** Joanna Danielewicz, Guillaume Girier, Anton Chizhov, Mathieu Desroches, Juan-Manuel Encinas, Serafim Rodrigues

**Affiliations:** 1 Achucarro Basque Center for Neuroscience, Leioa, Bizkaia, Spain; 2 BCAM Basque Center for Applied Mathematics, Bilbao, Bizkaia, Spain; 3 Ioffe Institute, St.-Petersburg, Russia; 4 Inria Branch of the University of Montpellier, Montpellier, France; 5 University of the Basque Country (UPV/EHU), Leioa, Bizkaia, Spain; 6 Ikerbasque, The Basque Science Foundation, Bilbao, Bizkaia, Spain; Indiana University Purdue University Indianapolis, UNITED STATES OF AMERICA

## Abstract

In response to prolonged depolarizing current steps, different classes of neurons display specific firing characteristics (i.e., excitability class), such as a regular train of action potentials with more or less adaptation, delayed responses, or bursting. In general, one or more specific ionic transmembrane currents underlie the different firing patterns. Here, we sought to investigate the influence of artificial sodium-like (Na channels) and slow potassium-like (KM channels) voltage-gated channels conductances on firing patterns and transition to depolarization block (DB) in Dentate Gyrus granule cells with dynamic clamp - a computer-controlled real-time closed-loop electrophysiological technique, which allows to couple mathematical models simulated in a computer with biological cells. Our findings indicate that the mimicked extra Na/KM channels significantly affect the firing rate of low-frequency cells, but not that of high-frequency cells. Moreover, we have observed that 44 percent of recorded cells exhibited what we have called a “flipping” behavior. This means that these cells were able to overcome the DB and generate trains of action potentials at higher current injection steps. We have developed a mathematical model of “flipping" cells to explain this phenomenon. Based on our computational model, we conclude that the appearance of “flipping" is linked to the number of states for the sodium channel of the model.

## Introduction

Depolarization block - a silent state that occurs when a neuron receives excessive excitation - is a very important feature regarded to have pathological relevance for some brain disorders, including epilepsy and schizophrenia [1–3]. Furthermore, depolarization block in dopaminergic neurons was suggested to explain the therapeutic action of antipsychotic drugs [3]. Among all the mechanisms responsible for transition into depolarization block, the inactivation of voltage-gated sodium channels is believed to play a key role [4–6]. Subsequently, it has been shown that decreasing the sodium conductance pharmacologically causes dopamine neurons to go into DB with lower maximal frequencies at lower values of applied current, whereas augmenting this conductance with the dynamic clamp has the opposite effect [6,7]. Dynamic clamp, a term for the various combinations of software and hardware that simulate these conductances, has proven to be a valuable tool for electrophysiologists to study different excitability classes of cells [8].

The dentate gyrus (DG) is the input gate to the mammalian hippocampal formation and it has been implicated in spatial navigation, response decorrelation, pattern separation and engram formation. Continual neurogenesis in the adult dentate gyrus produces new granule cells (GCs) that integrate into the hippocampal circuit by establishing synapses with existing neurons [9–12]. Granule cells are the prominent neuronal subtype within the DG, and they have been studied extensively from the perspective of their intrinsic response properties. GCs are characterized by their peculiar delayed and heterogeneous maturation. Most of them (85%) are generated postnatally. From the primary dentate matrix, neural precursors migrate to the dentate gyrus between embryonic day 10 and 14 where they differentiate into neurons [13,14]. Neurogenesis reaches a peak at the end of the first postnatal week and is largely completed toward the end of the first postnatal month [15]. Interestingly, the dentate gyrus retains the capability to give rise to new neurons throughout life, although at a reduced rate [16,17]. In adulthood, after being generated in the subgranular zone, immature GCs are incorporated into pre-existing circuits, thus contributing to improve several brain functions including learning and memory processes. During a transient period of maturation, new GCs exhibit intrinsic and synaptic properties distinct from mature GCs, potentially underlying the contribution of neurogenesis to memory encoding [12,18–23].

The vast repertoire of electrical activity displayed by neurons is the result of membrane-bound ion channels, each producing a distinct conductance that facilitates current flux through the membrane. These conductances may be static, or their magnitudes may be voltage- or ligand-dependent. The intrinsic firing properties and ionic conductances in GCs are thought to reflect their developmental stage and maturation level [18,24,25]. Among DG granule cells, input resistance (*R*_*i*_), threshold current (*I*_*thr*_), and firing patterns have been used as signatures of the degree of maturation and circuitry integration. During the first few days, postmitotic neurons remain in the proliferative subgranular zone and display very high input resistance (several gigaohms), because of the low density of K-channels in the plasma membrane. Immature GCs also express voltage-dependent Na- and K-channels at a low density. Thus, depolarizing current steps elicit “immature" action potential (single spikes with small amplitude and long duration) in current-clamp recordings [16,17,26]. Maturating GCs show a progressive decrease in input resistance and an increase in spike amplitude and frequency, suggesting that a deep rearrangement of voltage-operated and non-gated channels occurs at the same time. It has been observed that granule cells, expressing less mature phenotype, are reaching a maximum number of spikes with current steps of moderate intensity. At current injections of high intensity, these cells are lacking sustained firing, and action potentials (APs) become progressively smaller in amplitude, eventually entering depolarization block. On the other hand GCs with more mature phenotype, are firing throughout the entire range of current steps and exhibit firing rates proportional to the amplitude of the injected current [27,28]. Based on that, we believe that the entry into depolarization block could be another important electrophysiological feature of granule cells that can be useful for distinguishing the level of maturation of these neurons.

In this study, we focused on exploring firing patterns and the transition to depolarization block of granule cells in the dentate gyrus by using dynamic-clamp electrophysiological recordings. We applied dynamic-clamp recordings to explore the diversity of neurons and to tackle their excitability by adding artificial sodium-like or potassium-like voltage-gated channels. This approach allowed us to describe for the first time a new electrophysiological phenomenon that we have called “flipping”.

## Materials and methods

### Animals and treatment

Four- to six-week old C57BL/6 mice were used for all procedures. Mice were housed at constant humidity and temperature with a 12-h light/dark cycle with food ad libitum. The use of animals for experimentation and the experimental procedures are included in the approved protocols M20_2022_129 (2022-2025); M20_2022_130 (2022-2025) that have been reviewed and approved by the ethics (CEID and CEIAB) committees of the UPV/EHU and the Diputación Foral de Bizkaia.

### Preparation of brain slices

Mice were anestetized with isoflurane and decapitated. Their brains were quickly removed and placed in ice-cold artificial cerebrospinal fluid (ACSF) containing (in mM): 92 NMDG, 2.5 KCl, 0.5 CaCl_2_, 10 MgSO_4_, 1.25 NaH_2_PO_4_, 30 NaHCO_3_, 20 HEPES, 5 Na-ascorbate, 3 Na-pyruvate, 2 Thiourea and 25 D-glucose (pH 7.3 – 7.4; 300 – 310 mOsm) and bubbled with the mixture of 95% O2 – 5% CO2. Coronal slices (thickness = 250 μm) containing DG were cut by using a vibrating microtome (Leica VT1000). Slices were stored submerged first in 32 ^°^C and later in room temperature for recovery.

### Electrophysiology

After 1-1.5 h, individual slices were placed in the recording chamber mounted on the stage of a Scientifica microscope with 40x water immersion lens and superfused at 3 ml/min with warm (33 ± 0.5 ^°^C), modified ACSF of the following composition (in mM): 124 NaCl, 4.5 KCl, 1.25 NaH_2_PO_4_, 26 NaHCO_3_, 1 MgSO_4_ * 7 H_2_O, 1.8 CaCl_2_, and 10 D-glucose (pH 7.3-7.4; 300-310 mOsm), bubbled with the mixture of 95% O2 – 5% CO2. Recording micropipettes were pulled from borosilicate glass capillaries (Science Products) using the PC-100 Nareshige puller. The pipette solution contained (in mM): 125 K-gluconate, 20 KCl, 2 MgCl_2_, 10 HEPES, 4 Na_2_-ATP, 0.4 Na-GTP, 5 EGTA (pH 7.3-7.4; 295-305 mOsm). Pipettes had open tip resistances of approx. 7-9 MΩ. The calculated liquid junction potential using this solution was approx. 13.1 mV, and data were corrected for this offset. Signals were recorded using Axon MultiClamp 700B amplifier (Molecular Devices), filtered at 2 kHz, and digitized at 20 kHz using Digidata 1550A (Molecular Devices) interface and Clampex 10 software (Molecular Devices, USA).

Cell access was obtained in the voltage-clamp mode and Resting Membrane Potential (RMP) was measured immediately upon break-in in the current-clamp mode by setting the clamp current equal to zero. In order to understand better the functional role of ion channels in shaping the electrical activity and entry into depolarization block, granule cells were recorded under dynamic-clamp conditions.

### Dynamic clamp

For recordings we used the dynamic clamp technique with the help of parallel acquisition at the second computer. In our specific setup, a second computer was connected to the MultiClamp 700B amplifier via the NI DAQ PCI-6221-37pin (National Instruments, Austin, TX). The acquisition time set at our NI-DAQ-6221 was 33kHz. We employed a dynamic-clamp setup with open-source software (Available at http://www.ioffe.ru/CompPhysLab/AntonV3.html). Our software allows for a flexible development environment, which is the key requirement for later enhancements of specific experimental protocols.

**Fig 1:**
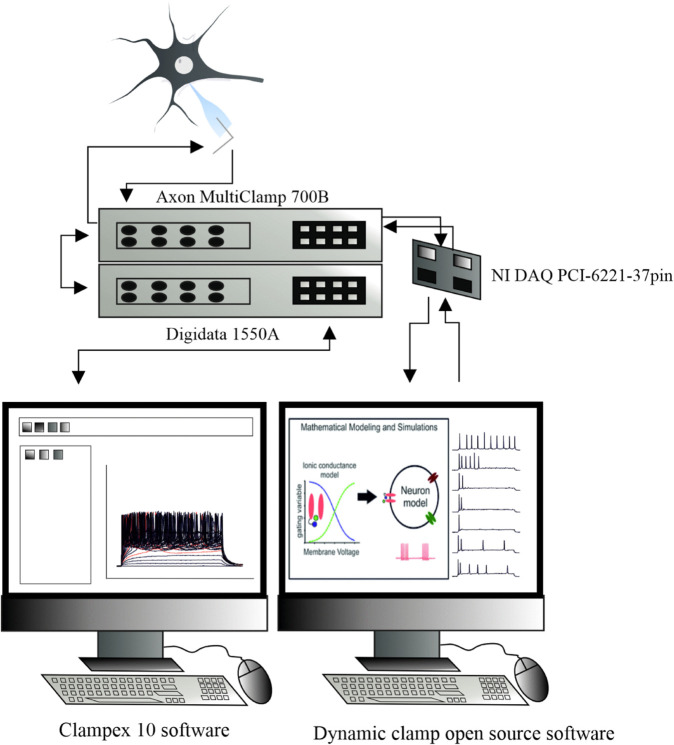
Dynamic-clamp experimental setup. The signal from the neuron is recorded by using Axon Multiclamp amplifier and Axon Digidata analog-digital converter and observed on the first computer in the Clampex 10 software package. The dynamic clamp loop is established by adding the second computer equiped with NI DAQ card connected directly to the Axon Multiclamp amplifier. The signal is observed by using dynamic clamp software (described in Materials and Methods section).

Once the two systems were connected, whole-cell recordings were performed in real-time and the current injected into the cell, *I*_*applied*_, was directly dependent on the measured voltage *V*. The firing characteristics of the recorded cells were assessed using intracellular injections of rectangular current pulses of increasing amplitude (range:- 200 pA to + 1200 pA; duration: 500 ms) and f-I curves (firing rate vs injected current) were constructed. With dynamic clamp, we mimicked additional channels and assessed several parameters, like conductance, half-maximum location of the activation and inactivation functions of the ion channels responsible for general electrical activity and entry into DB of the recorded cells. To describe the additional currents, we employed conventional approximations such as Hodgkin-Huxley ones for potassium channels, and Markovian model for sodium channels. Only cells with stable access resistance were accepted for the data analysis. Our dynamic-clamp software is compatible with the standard Clampfit software which was used in order to calculate input resistance and membrane time constant tau. From each cell traces obtained in dynamic-clamp current clamp mode in control conditions, before “adding" sodium and potassium channels, were selected and exported as abf files supported by Clampfit. In Clampfit all traces below the rheobase were selected and the input resistance was calculated as the mean resistance from selected sweeps. In order to obtain tau one sweep per each cell was selected. Tau was calculated by fitting an exponential function with Chebyshev polynomials. A schematic of our experimental setup is presented in Fig 1.

In the experiments where additional shunting was mimicked, the injected current was calculated as a linear function of the recorded membrane potential *V*(*t*):

$$I_{applied}(V,t)=u(t)-s(t)(V(t)-V_{us}),$$ (1)

where *u*(*t*) and *s*(*t*) are step functions of time. In our experiments and simulations, the reference membrane potential $V_{us}$ was chosen to be equal to −60mV. However, we note that a particular choice of $V_{us}$ is not essential for the results, because values of *u*(*t*) and *s*(*t*) can be re-calculated for any alternative value of $V_{us}$ through their values for $V_{us}=-60\;\text{mV}$. The input signal *s*(*t*) can be interpreted as an extra, mimicked conductance; and *u*(*t*) is a voltage-independent current as if measured at the fixed voltage level $V_{us}$. Note as well that *u*(*t*) and *s*(*t*) can be re-interpreted in terms of excitatory and inhibitory conductances *G*_*E*_(*t*) and *G*_*I*_(*t*) with the corresponding constant reversal potentials $V_{E}$ and $V_{I}$, respectively:


$$I_{applied}(V,t)=u(t)-s(t)(V-V_{us})=G_{E}(t)(V_{E}-V)+G_{I}(t)(V_{I}-V),$$

that is,


$$u(t)=G_{E}(t)(V_{E}-V_{us})+G_{I}(t)(V_{I}-V_{us}),\;s(t)=G_{E}(t)+G_{I}(t).$$

Note also that additional synaptic components with voltage-independent conductances would not change the number of input signals, thus *u*(*t*) and *s*(*t*) fully represent any set of such synaptic channels. For instance, introducing another synaptic type with $G_{E^{\prime }}$ and $V_{E^{\prime }}$ would give


$$u(t)=G_{E}(t)(V_{E}-V_{us})+G_{E^{\prime }}(t)(V_{E^{\prime }}-V_{us})+G_{I}(t)(V_{I}-V_{us}),\;s(t)=G_{E}(t)+G_{E^{\prime }}(t)+G_{I}(t).$$

Hence, the variety of *u*(*t*) and *s*(*t*) provides all physiologically meaningful variety of *I*_*applied*_ that mimic the total synaptic current, under the assumption of the voltage independence of synaptic conductances and reversal potentials. In the present work, we consider only stepwise time-dependence of functions of *u*(*t*) and *s*(*t*) of various amplitudes.

By using the dynamic-clamp technique, we were able to mimic the addition of “extra" voltage-gated sodium and potassium channels. We are aware of the fact that the dynamic clamp just artificially mirrors the effects of additional ion channels, but it is not actually adding such channels in the neuronal membrane like with other experimental approaches that might directly up- or down-regulate the expression of a particular ion channel in the membrane. However, in this manuscript we will be using the somewhat loose terminology of “added" or “extra" channels in order to keep the presentation simpler and clearer for the readers. In order to establish the values for conductances of extra Na channels and the composition and values for conductances of K channels trials aimed at eliciting a similar and optimal responses in each individual cell were run. For majority of our cells (10 out of 17) the “best” composition was the addition of Na channel with the conductance set at 50nS and KM channel with the conductances set at 50 nS and 20 nS depending on the response. In the response we focused on the number of action potentials elicited by the current step at relatively low values from 40 to 80 pA. Not all neurons were generating a similar response with these settings, therefore the values of conductances for both Na and K were adjusted. Out of the remaining 7 cells 4 required the Na conductance at 100 nS, 2 cells required the Na conductance at 10 nS and 1 cell required the Na conductance at 200 nS. Vast majority (11 out of 17) of cells were recorded with the addition of only KM channels, however not for all of the recorded neurons, therefore the composition of the different K channels was also adjusted. 3 out of the remaining 6 cells were recorded with only KA channel and the remaining 3 cells required the addition of both KM and KA channels.

In the experiments with additional sodium and potassium channels, the injected current was as follows:

$$I_{applied}(V,t)=I_{Na}(V,t)+I_{DR}(V,t)+I_{A}+I_{KM}(V,t)+u(t)-s(t)(V(t)-V_{us}),$$ (2)

where *I*_*Na*_, *I*_*DR*_, *I*_*A*_, and *I*_*KM*_ were calculated according to ordinary differential equations (ODEs) coming from a mathematical model. In our simulations and dynamic clamp experiments with additional channels we used the same approximations accordingly.

### Computational modeling

We have combined multi-timescale mathematical modeling for excitability framework developed in our previous work [29] and conductance-based modeling.

The model’s equations are defined as follows:

$$C\frac{dV}{dt}=g_{L}(V-V_{L})-I_{Na}-I_{DR}-I_{A}-I_{KM}+u(t)-s(t)(V-V_{us})$$ (3)

Approximating formulas for the currents *I*_*Na*_, *I*_*DR*_ and *I*_*A*_ are taken from [30]. The voltage-dependent potassium current *I*_*DR*_, which provides fast spike repolarization, is defined as:

$$I_{DR}(V)={\bar{g}}_{DR}\;n\;y_{K}(V-V_{K}),$$ (4)

$$\frac{dn}{dt}=\frac{n_{\infty }(V)-n}{\tau_{n}(V)},$$ (5)

$$\frac{dy_{K}}{dt}=\frac{{y_{K}}_{\infty }(V)-y_{K}}{\tau_{y}(V)},$$ (6)

$$\tau_{n}=\frac{1}{\alpha_{n}+\beta_{n}}+0.8\;\text{ms},\;n_{\infty }=\frac{\alpha_{n}}{\alpha_{n}+\beta_{n}}\;\alpha_{n}=0.17\cdot e^{(V+5)\cdot 0.090}\;{\text{ms}}^{-1},\;\beta_{n}=0.17\cdot e^{(V+5)\cdot 0.022}\;{\text{ms}}^{-1},\;\tau_{y}=300\;\text{ms},\;{y_{K}}_{\infty }=\frac{1}{1+e^{(V+68)\cdot 0.038}}\;$$ (7)

The voltage-dependent potassium current *I*_*A*_, that provides spike repolarization, is defined as:

$$I_{A}(V)={\bar{g}}_{A}n_{A}^{4}l_{A}^{3}(V-V_{K}),$$ (8)

$$\frac{dn_{A}}{dt}=\frac{n_{A\infty }(V)-n_{A}}{\tau_{n_{A}}(V)},$$ (9)

$$\frac{dl_{A}}{dt}=\frac{l_{A\infty }(V)-l_{A}}{\tau_{l_{A}}(V)},$$ (10)

$$\tau_{n_{A}}=\frac{1}{\alpha_{n_{A}}+\beta_{n_{A}}}+1\;\text{ms},\;n_{A\infty }=\frac{\alpha_{n_{A}}}{\alpha_{n_{A}}+\beta_{n_{A}}}\;\alpha_{n_{A}}=0.08\cdot e^{(V+41)\cdot 0.089}\;{\text{ms}}^{-1},\;\beta_{n_{A}}=0.08\cdot e^{(V+41)\cdot 0.016}\;{\text{ms}}^{-1},\;\tau_{l_{A}}=\frac{1}{\alpha_{l_{A}}+\beta_{l_{A}}}+2\;\text{ms},\;l_{A\infty }=\frac{\alpha_{l_{A}}}{\alpha_{l_{A}}+\beta_{l_{A}}},\;\alpha_{l_{A}}=0.04\cdot e^{-(V+49)\cdot 0.11}\;{\text{ms}}^{-1},\;\beta_{l_{A}}=0.04\;{\text{ms}}^{-1}$$ (11)

The voltage-dependent potassium current *I*_*KM*_, that provides medium adaptation, is defined as:

$$I_{KM}(V)={\bar{g}}_{KM}n_{KM}^{2}l_{KM}(V-V_{K}),$$ (12)

$$\frac{dn_{KM}}{dt}=\frac{n_{KM\infty }(V)-n_{KM}}{\tau_{KM}(V)},$$ (13)

$$\frac{dl_{KM}}{dt}=\frac{l_{KM\infty }(V)-l_{KM}}{1000\text{ms}},$$ (14)

$$\tau_{KM}=\frac{1}{\alpha_{KM}+\beta_{KM}}+8\;\text{ms},\;n_{KM\infty }=\frac{\alpha_{KM}}{\alpha_{KM}+\beta_{KM}}\;\alpha_{KM}=0.003\cdot e^{(V+45)\cdot 0.14}\;{\text{ms}}^{-1},\;\beta_{KM}=0.003\cdot e^{(V+45)\cdot 0.094}\;{\text{ms}}^{-1},\;l_{KM\infty }=\frac{1}{1+e^{(V+40)/5}}$$ (15)

The voltage-dependent sodium current *I*_*Na*_ was approximated by the 4-state Markov model as proposed in [30]. The model with Hodgkin-Huxley-like approximations of potassium channels and Markovian model of sodium channels was initially used for a multicompartmental neuron; in [31] it was applied to one-compartmental neuron. The main advantage of the Markovian model is that it provides more quantitatively accurate approximation of the “window current" and the shape of the spike, as well as the non-overestimated effect of potassium currents at repolarization phase of a spike, due to which, in contrast to canonical Hodgkin-Huxley approximation, this model reproduces a pure sodium spike with a realistic shape in the conditions of potassium channel blockade. The model includes one open state (1), one inactivated state (2), and two closed states (3 and 4). Only the transitions 1→2, 2→3, 3→4, 3→1 and 4→1 are effective. The closed states help to introduce variable threshold, while keeping both transitions stiff enough to provide a kinky spike shape. Among the variety of known Markovian models of sodium currents with the number of states up to a few tens of them [32], this model is a minimal one that introduces an only alternative (second) closed state. The Markovian model is as follows:

$$I_{Na}(V)={\bar{g}}_{Na}x_{1}(V-V_{Na}),$$ (16)

$$x_{1}+x_{2}+x_{3}+x_{4}=1,$$ (17)

$$\frac{dx_{i}}{dt}=\sum_{j=0,j\neq 1}^{4}A_{j,i}x_{j}-x_{i}\sum_{j=0,j\neq 1}^{4}A_{i,j}\text{ with }i=1,2,3,$$ (18)

$$A_{1,2}=3\text{ ms },A_{1,3}={f\;}_{1}^{1,3}(V),A_{1,4}={f\;}_{1}^{1,4}(V),\;A_{2,1}=0,A_{2,3}={f\;}_{2}^{2,3}(V),A_{2,4}=0,\;A_{3,1}={f\;}_{1}^{3,1}(V),A_{3,2}=0,A_{3,4}={f\;}_{2}^{3,4}(V),\;A_{4,1}={f\;}_{1}^{4,1}(V),A_{4,2}=0,A_{4,3}=0,\;$$ (19)

$${f\;}_{1}^{i,j}(V)={\left\{\tau_{min}^{i,j}+\frac{1}{e^{(V-V_{1/2}^{i,j})/k^{i,j}}}\right\}}^{-1}\;{f\;}_{2}^{i,j}(V)=\{\tau_{min}^{i,j}+[(\tau_{max}^{i,j}-\tau_{min}^{i,j}{)}^{-1}+e^{(V-V_{1/2}^{i,j})/k^{i,j}}{]}^{-1}{\}}^{-1},\;\tau_{min}^{1,3}=1/3\text{ms},V_{1/2}^{1,3}=-51\text{mV},k^{1,3}=-2\text{mV},\;\tau_{min}^{1,4}=1/3\text{ms},V_{1/2}^{1,4}=-57\text{mV},k^{1,4}=-2\text{mV},\;\tau_{min}^{2,3}=1\text{ms},V_{1/2}^{2,3}=-53\text{mV},k^{2,3}=-1\text{mV},\tau_{max}^{2,3}=100\text{ms},\;\tau_{min}^{3,1}=1/3\text{ms},V_{1/2}^{3,1}=-42\text{mV},k^{3,1}=1\text{mV},\;\tau_{min}^{3,4}=1\text{ms},V_{1/2}^{3,4}=-60\text{mV},k^{3,4}=-1\text{mV},\tau_{max}^{3,4}=100\text{ms},\;\tau_{min}^{4,1}=1/3\text{ms},V_{1/2}^{4,1}=-51\text{mV},k^{4,1}=1\text{mV},\;$$ (20)

A reduced, 3-state approximation of the voltage-dependent sodium current *I*_*Na*_, was considered as follows:

$$I_{Na}(V)={\bar{g}}_{Na}x_{1}(V-V_{Na}),$$ (21)

$$x_{1}+x_{2}+x_{3}=1,$$ (22)

$$\frac{dx_{i}}{dt}=\sum_{j=0,j\neq 1}^{3}A_{j,i}x_{j}-x_{i}\sum_{j=0,j\neq 1}^{3}A_{i,j}\text{ with }i=1,2,$$ (23)

$$A_{1,2}=3\text{ ms },A_{1,3}={f\;}_{1}^{1,3}(V),\;A_{2,1}=0,A_{2,3}={f\;}_{2}^{2,3}(V),\;A_{3,1}={f\;}_{1}^{3,1}(V),A_{3,2}=0,\;$$ (24)

$${f\;}_{1}^{i,j}(V)={\left\{\tau_{min}^{i,j}+\frac{1}{e^{(V-V_{1/2}^{i,j})/k^{i,j}}}\right\}}^{-1}\;{f\;}_{2}^{i,j}(V)=\{\tau_{min}^{i,j}+[(\tau_{max}^{i,j}-\tau_{min}^{i,j}{)}^{-1}+e^{(V-V_{1/2}^{i,j})/k^{i,j}}{]}^{-1}{\}}^{-1},\;\tau_{min}^{1,3}=1/3\text{ms},V_{1/2}^{1,3}=-51\text{mV},k^{1,3}=-2\text{mV},\;\tau_{min}^{2,3}=1\text{ms},V_{1/2}^{2,3}=-53\text{mV},k^{2,3}=-1\text{mV},\tau_{max}^{2,3}=100\text{ms},\;\tau_{min}^{3,1}=1/3\text{ms},V_{1/2}^{3,1}=-51\text{mV},k^{3,1}=1\text{mV}\;$$ (25)

This approximation was obtained from the 4-state model by omitting the second closed state indexed “4" and setting the threshold value of the transition from closed to open state, $V_{1/2}^{3,1}$, equal to $V_{1/2}^{4,1}$.

### Statistical analysis

Statistical analysis was performed using the aforementioned dynamic-clamp open-source software Visualizator and GraphPad Prism (https://www.graphpad.com/). Data were presented as *mean* ± *SEM* and statistical significance was analyzed using a one–way ANOVA and one-tailed t-test with Welch’s correction. Post hoc analysis for ANOVA was conducted by using Tukey’s test. The level of statistical significance was set at *p* < 0.05. All data sets were tested for deviation from the normal distribution (Kolmogorov–Smirnov’s test). Heatmaps representing firing rates were created in Tecplot software (Tecplot Inc.).

## Results

### Intrinsic properties, firing pattern and depolarization block

**Fig 2:**
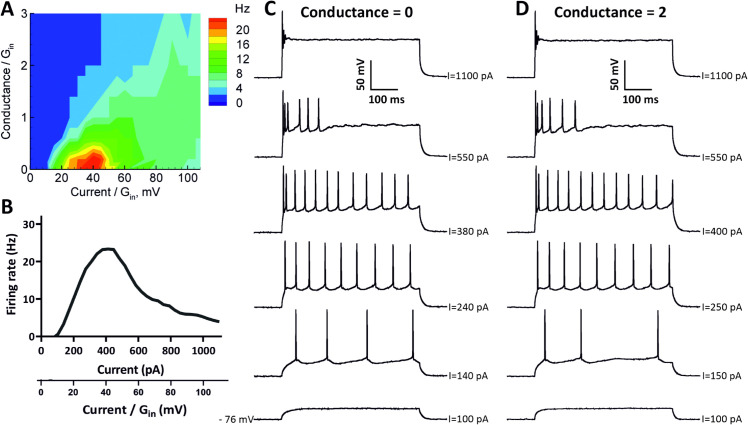
Assessment of firing rate in granule cells. (A) Firing rate as the number of APs per stimulation time interval (500ms) for a representative neuron as a function of injected current *u* and extra conductance *s*, both scaled by the input conductance $G_{in}$=9.9nS. Cell 21/C4. (B) Firing rate vs injected current steps ((*f*, *I*) curve) for cell 21/C4. (C) Representative responses of 21/C4 cell to sub- and suprathreshold depolarizing current pulses showing spiking pattern, entry into DB and full depolarization block without extra conductance (Conductance=0). (D) Representative responses of 21/C4 cell to sub- and suprathreshold depolarizing current pulses showing spiking pattern, entry into DB and full depolarization block under shunting conditions (Conductance=2).

We studied the firing patterns and the capabilities of granule cells for entering DB under dynamic-clamp conditions. The firing rate was assessed by constructing (*f*, *I*) curves for each individual neuron; see Fig 2B. Furthermore, the maximum frequency of action potentials was calculated as the number of spikes per stimulation time (500 ms).

Whereas in experimental conditions, synaptic currents are often represented by a voltage-independent currents, in natural conditions, they are always voltage-dependent as the sum over all types of synapses of the products of the synaptic conductances and the driving forces, the differences of membrane and reversal potentials. Assuming synaptic conductances to be voltage-independent, this sum can be represented as a linear function of the membrane potential, with a total synaptic conductance *s* as a coefficient and a free term *u* (current). In this assumption, we approximate an arbitrary synaptic current in the form of Eq 1 with two voltage-independent signals *u*(*t*) and *s*(*t*). Therefore, we have performed our recordings taking conductance *s* into account, and we have presented the firing rate as a two-parameter function, which is more informative than traditional (*f*, *I*) curves, revealing a full domain of excitability in the plane of those two main signals *u* and *s*. In particular, because DB depends not only on the injected current but also on the extra (synaptic) conductance (additional shunting), we measured the dependence of firing rate versus current and conductance (Fig 2A), thus revealing the full domain of spiking in the plane corresponding to these input parameters.

**Fig 3:**
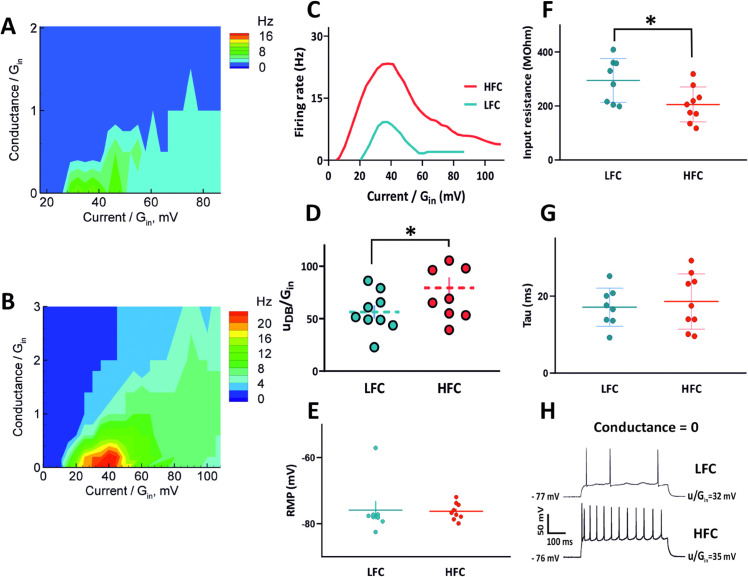
Comparison between two subpopulations of recorded cells. (A) Firing rate for a representative neuron from LFC group as a function of injected current and extra conductance, both scaled by the input conductance $G_{in}$=3.5nS. Cell 21/C1. (B) Firing rate for a representative neuron from HFC group as a function of injected current and extra conductance, both scaled by the input conductance $G_{in}$=9.9nS. Cell 21/C4. (C) (*f*, *I*) curves for representative neurons from LFC and HFC. (D) LFC are entering depolarization block earlier than HFC. (E,G) No significant differences were found in resting membrane potential (RMP) and membrane time constant tau. (F) Neurons from LFC group have significantly higher input resistance than HFC. (H) Representative responses of LFC (top) and HFC (bottom) to suprathreshold depolarizing current pulse.   indicate statistical significance p < 0.05 ANOVA.

The entry into DB was defined when the recorded cells started to generate action potentials with half of the maximum spiking frequency. We then measured the quantity $u_{DB}/G_{in}$, where *u*_*DB*_ is the value of *u* leading to DB, and *G*_*in*_ is the input conductance of neuron at the resting state, evaluated from responses to current step injection. Based on firing characteristics, specifically maximum frequency of the generated action potentials, we have observed that recorded cells can be divided into two main groups:

1) LFC (low-frequency cells), generating action potentials with a maximum frequency lower than 12 Hz (8 cells out of 17; Fig 3A, 3C), and

2) HFC (high-frequency cells), generating action potentials with a frequency higher than 12 Hz (9 out of 17 cells; Fig 3B, 3C).

The maximum spiking frequency reached by LFC was significantly lower when compared to HFC (7.5 ± 1.4 Hz vs 22.9 ± 2.1 Hz, *p* < 0.0001; see Fig 3).

In LFC group, the firing rate decreased for $u_{DB}/G_{in}$ bigger than 53.55 ± 6.48 mV due to depolarization block and it was significantly lower than in HFC group (79.35 ± 10.09 mV; p=0.0251;F=2.730;t=2,152;df=13.36; see Fig 3D), which indicates that LFC are entering into DB earlier than HFC. We have compared basic intrinsic properties of recorded cells, such as RMP, input resistance and tau. In LFC group input resistance is significantly higher than in HFC (LFC 294.8 ± 28.77 MOhm; HFC 202.1 ± 17.58 MOhm; *p* = 0.0026;F(3,30)=59.94; ANOVA; Fig 3F). No significant differences were found in resting membrane potential and tau (LFC RMP −75.89 ± 2.754 mV; HFC RMP −76.26 ± 0.874 mV; *p* > 0.999; F(3,30)=67.20; LFC tau 17.11 ± 1.756 ms; HFC tau 18.59 ± 2.395 ms; *p* > 0.999; F(3,30)=59.9; ANOVA; Fig 3E, 3G). Therefore, we suggest that the maximum firing rates of different neurons are mainly related to the current critical for entering DB.

### Effect of extra channels

**Fig 4:**
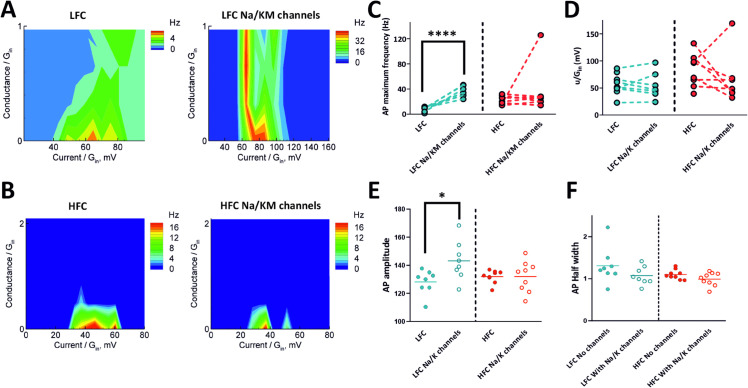
Effects of additional sodium and slow potassium channels on firing rate and entry into depolarization block. (A) Firing rate for a representative LFC without (left) and with additional Na/KM channels (right; $g_{Na}$=200 nS, $g_{KM}$=200 nS). (B) Firing rate for a representative HFC without (left) and with additional Na/KM channels (right; $g_{Na}$=10 nS, $g_{KM}$=10 nS). (C) Maximum spiking frequency was significantly increased in LFC, but not in HFC. (D) Addition of Na/KM channels does not influence the $u/G_{in}$ parameter. (E) Addition of Na/KM channels increases the amplitude of action potential only in LFC group. (F) AP duration is not affected by extra Na/KM channels. (**** indicate statistical significance *p* < 0.0001 ANOVA, * indicates statistical significance *p* < 0.05 ANOVA).

We studied whether additional sodium and potassium channels would significantly affect both firing rates and entry into the depolarization block of the recorded GCs. For the sodium channels, we used the 4-state Markov model described in the Methods section. Among potassium channels, majority of neurons were recorded only with the extra KM-type channel. It is slower than the DR-channel and the A-channel, which provides more effective repolarization after a spike and thus de-inactivation of sodium channels [33], both natural and mimicked ones. KM-channels support spiking and enlarge the range of injected currents that provide spike generation. Conductance of “extra" channels was adjusted individually for every cell in accordance with their sensitivity, the criteria of this adjustment was to elicit a visible effect. As a result, LFC started showing much higher values of firing rate and the maximum frequency of generated action potentials was significantly higher when additional Na/KM channels were introduced (without extra channels: 7.5 ± 1.4 Hz, vs with Na/KM channels: 36 ± 3 Hz; *p* < 0.0001; *F* = 4.5; *t* = 8; *df* = 8; Fig 4A, 4C). However, adding Na/KM channels did not significantly affected the $u_{DB}/G_{in}$ parameter, therefore the entry into DB of LFC has not been influenced (without extra channels: 56 ± 6 mV vs with Na/KM channels: 54 ± 9 mV; *p* = 0.4; *F* = 1.6; *t* = 0.17; *df* = 11; Fig 4D). In the HFC group, after adding extra Na/KM channels, a change to the (*f*, *I*) curve previously observed in the LFC was present only in 1 out of 9 recorded cells. In the remaining HFC, additional Na/KM channels did not affect firing rate (Fig 4B, 4C). Overall, the mean maximum frequency of generated action potentials in HFC was not significantly altered by the addition of Na/KM channels (without extra channels: 23 ± 2 Hz, vs with Na/KM channels: 36 ± 13 Hz; *p* = 0.34; *F* = 30; *t* = 1.0; *df* = 7.5; Fig 4B, 4C). In the HFC group, after adding Na/KM channels, cells tend to enter DB block earlier than without additional channels as the value of the $u_{DB}/G_{in}$ parameter is smaller, however this shift is not statistically significant (without extra channels: 82 ± 11 mV, vs with Na/KM channels: 66 ± 18 mV; *p* = 0.23; *F* = 2.3; *t* = 0.76; *df* = 10; Fig 4D). Because the amplitude of the action potential is mainly dependent on the influx of Na+, the addition of extra Na/KM channels could affect this parameter, therefore we have compared action potential amplitude and duration measured as half width, before and after the addition of Na/KM channels. AP amplitude was measured as the total change in voltage from the resting state (RMP) to the peak of the action potential (S1A Fig). In LFC group addition of Na/KM channels significantly increased the amplitude of spikes (without extra channels 128.2 ± 3.052 mV vs with Na/KM channels 143.2 ± 4.905 mV; *p* = 0.0106;F(3,30)=3.413; ANOVA) and in AP peak (without extra channels 52.46 ± 1.856 mV vs with Na/KM channels 65.85 ± 3.817 mV; *p* = 0.004; *t* = 3.154;*df* = 11.58; F(8,8)=4.230; t-test with Welch’s correction; (S1B Fig), however the duration of action potential was not affected (without extra channels 1.309 ± 0.151 ms vs with Na/KM channels 1.073 ± 0.076 ms; *p* = 0.1404; F(3,30)=2.394; ANOVA). In HFC group neither the AP amplitude, the AP peak nor half width were affected by additional Na/KM channels (AP amplitude without extra channels 131.9 ± 1.525 mV vs with Na/KM channels 132.0 ± 3.620 mV; *p* > 0.999;F(3,30)=3.413; AP peak without channels 55.94 ± 1.254 ms vs with Na/KM channels 58.25 ± 3.171 ms;*p* = 0.2575;F(3,30)=2.394; AP half width without channels 1.101 ± 0.0382 ms vs with Na/KM channels 0.992 ± 0.0533 ms; *p* = 0.7263;F(3,30)=2.394; ANOVA). No significant changes in RMP after addition of extra channels in both groups were observed (S1C Fig). Moreover, the addition of Na/KM channels has no significant effect on the input resistance in LFCs (without extra channels 294.8 ± 28.77 MOhm vs with Na/KM channels 297.0 ± 35.32 MOhm; *p* = 0.481;*t* = 0.04859;*df* = 13.45; F(7,7)=1.507); t-test with Welch’s correction; (S1D Fig)). In HFC group we observed a trend of increased resistance, however the change has not reached statistical significance (without extra channels 202.1 ± 17.58 MOhm vs with Na/KM channels 267.9 ± 38.93 MOhm; *p* = 0.08;; *t* = 1.54;*df* = 8.35; F(6,6)=4.901); t-test with Welch’s correction; (S1D Fig)). We compared the input resistance between LFCs and HFCs after the addition of extra channels. The initial difference between these groups described in the previous section (without extra channels) is no longer present (LFC with Na/KM channels 297.0 ± 35.32 MOhm vs HFC with Na/KM channels 267.9 ± 38.93 MOhm; *p* = 0.295;; *t* = 0.554;*df* = 12.62; F(6,7)=1.063); t-test with Welch’s correction; (S1D Fig).

### “Flipping” cells

**Fig 5:**
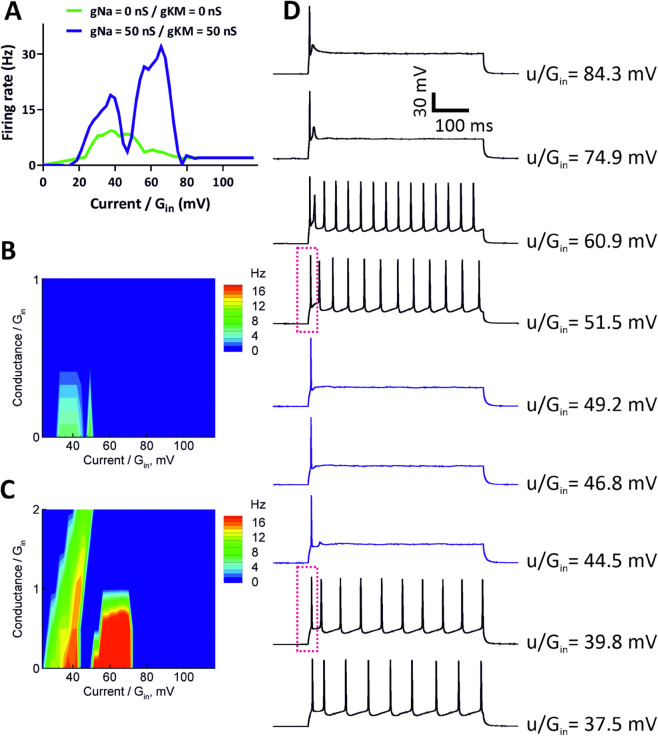
Example of the “flipping” phenomenon. (A) (*f*, *I*) curves of a representative cell exhibitng a pronounced “flip" in firing pattern induced by the addition of $g_{Na}$=50 nS, $g_{KM}$=50 nS channels (blue line). (B, C) Firing rate as a function of injected current and extra conductance, both scaled by the input conductance $G_{in}$=4.27nS without (B) and with (C) additional Na/KM channels. (D) Representative responses to a suprathreshold depolarizing current pulses. Traces in blue are representing first depolarization block. Red rectangles indicate first action potential in the traces before and after the “flip".

**Fig 6:**
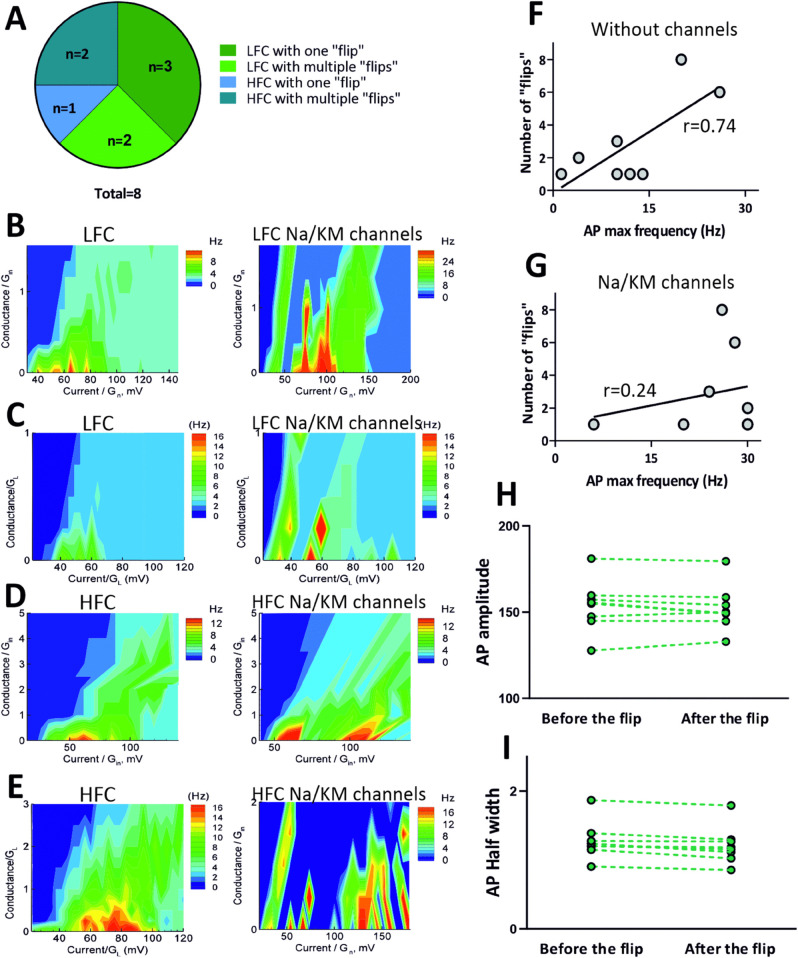
“Flipping” cells. (A) Division of “flipping” cells based on the number of “flips" and the initial firing rate (LFC vs HFC). (B) Firing rate of a representative LFC generating one “flip" after addition of $g_{Na}$=100 nS, $g_{KM}$=40 nS; Cell 21/C3. (C) Firing rate of a representative LFC generating multiple “flips" after addition of $g_{Na}$=50 nS, $g_{KM}$=50 nS; Cell 21/C6. (D) Firing rate of a representative HFC generating one “flip" after addition of $g_{Na}$=50 nS, $g_{KM}$=20 nS; Cell 21/C2. (E) Firing rate of a representative HFC generating multiple “flips" after addition of $g_{Na}$=50 nS, $g_{KM}$=50 nS; Cell 21/C7. (F) The number of “flips" is correlated with the maximum frequency of spikes generated in response to injected current step before the addition of Na/KM channels. (G) The number of “flips" is not correlated with the maximum frequency of spikes generated in response to injected current step after the addition of Na/KM channels. (H,I) No changes were observed in action potentials before and after the “flip".

While analyzing (*f*, *I*) curves from recorded neurons, we have observed an interesting phenomenon regarding depolarization block. In several granule cells, after adding Na/KM channels we observed a complex, non-gradual dependence of the firing rate onto injected current. While, in general, the initial entry into DB was not affected by additional Na/KM channels as there were no significant differences in $u_{DB}/G_{in}$ (as described in the previous section), 8 out of 18 recorded neurons expressed a very unique behavior in terms of their firing patterns. After reaching a full DB characterized as generating only 1 or 2 action potentials in response to injected current step, these cells were not able to maintain it, instead they “flipped" and started generating trains of spikes at larger injected current steps before finally reaching another DB. We called this phenomenon “flipping” (Fig 5). Interestingly, even within this small subpopulation of “flipping” cells we were able to observe two different types of “flipping” behavior. Half of the recorded cells were able to overcome the DB only once, meaning one large “flip" was present in their firing responses to current injection. However, the remaining half of the “flipping” neurons were able to produce multiple “flips" (2 to 8). In general, we observed that the majority (5 out of 8) of “flipping” neurons were previously assigned to the LFC group and within this subpopulation 2 cells were able to generate multiple “flips" (2 and 3 flips). The remaining 3 “flipping” cells belonged to HFC group and 2 of those cells were able to generate 6 and 8 “flips" in response to injected current step (Fig 6A). As the majority of “flipping” cells generating only one “flip" were previously assigned to the LFC group, we wondered whether the number of “flips" is correlated with the initial spiking frequency. We have observed a positive correlation between the number of “flips" and the maximum frequency of action potentials generated by the cell in control conditions, meaning without additional Na/KM channels (*r* = 0.74; *R*^2^ = 0.54; *p* = 0.037; Fig 6F), however there was no correlation between the number of generated “flips" and the maximum spiking frequency recorded with additional Na/KM channels present (*r* = 0.24; *R*^2^ = 0.055; *p* = 0.58; Fig 6G). We compared the amplitude and duration of action potential in “flipping" cells before and after the “flip". For the comparison we have chosen the first spike appearing in the trace before the “flip" and the first spike appearing in the trace right after as indicated by red rectangles in Fig 5D. No significant differences were observed in the AP amplitude and half width. (AP amplitude before the “flip" 153.6 ± 5.333 mV vs after the “flip 152.3 ± 4.709 mV; *p* > 0.999;F(3,28)=606.1; AP half width before the “flip" 1.279 ± 0.0971 ms vs after the “flip" 1.211 ± 0.0964 ms; *p* > 0.999;F(3,28)=606.1; ANOVA; Fig 6H, 6I). Because “flipping” is a phenomenon that we have never come across before, we wondered what could be the possible mechanism behind it. For this purpose, we have designed and analyzed a computational model dedicated to this phenomenon.

### “Flipping” in a computational model

**Fig 7:**
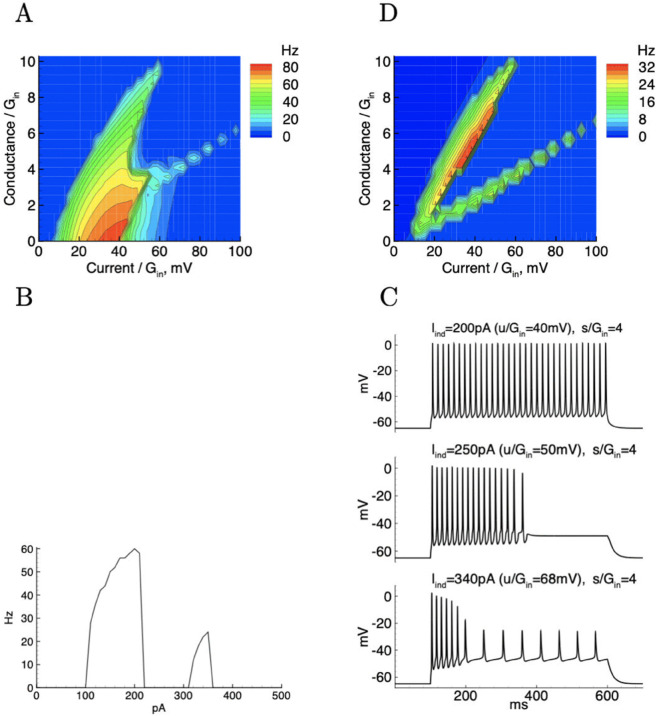
Simulations of a neuron model with Na- and K-channels. $g_{Na}$=228nS, $g_{DR}$ = 500nS, Gin = 5nS, C = 70pF, VL = −65mV, Vus = −60mV, VK = −70mV, VNa = 65mV. A: f-u-s dependence; B: (f, I) curves for s/Gin=4; C: spike trains. D: Reduced model with only Na- and leaky channels (gK = 0).

In order to analyze the “flipping” effect, we simulated a neuron model with a Na- and K-channels. In this model, the “flipping” is observed only for nonzero extra conductance *s*. The excitation domain (Fig 7A) has a “horn" on the right. Hence, the (*f*, *I*) curve shows a gap (Fig 7C), which is due to the “flipping”. In terms of spike trains (Fig 7B), this gap is revealed as a cessation of spiking for intermediate currents, and constant spiking in response to smaller and larger currents. At large currents, however, the amplitude of spikes is different, which is explained by a different sequence of transitions undergone by the sodium channels between the states of the Markov model. While during weak stimulus the hyperpolarization between spikes is stronger and the channels pass through low- and high-threshold states, for larger current the neuronal membrane is always depolarised between spikes and the channels pass only through high-threshold states. Amplitude of action potentials is significantly reduced for larger current, as seen from (Fig 7C). This recruitment of different channel states in different regimes causes the “flipping” behavior.

Blockade of potassium channels in the model leads to shrinkage of the excitation domain (Fig 7D), which is visibly narrower than in (Fig 7A), however the “horn" on the right remains indicating the presence of “flipping”. In the reduced model with only Na- and leaky channels present, the “flipping” is observed in a wider range of extra conductances. We note that the spike generation without K-channels, observed in experiments, is possible due to the Na-channel approximation from [30].

### Bifurcation analysis of “flipping”

**Fig 8:**
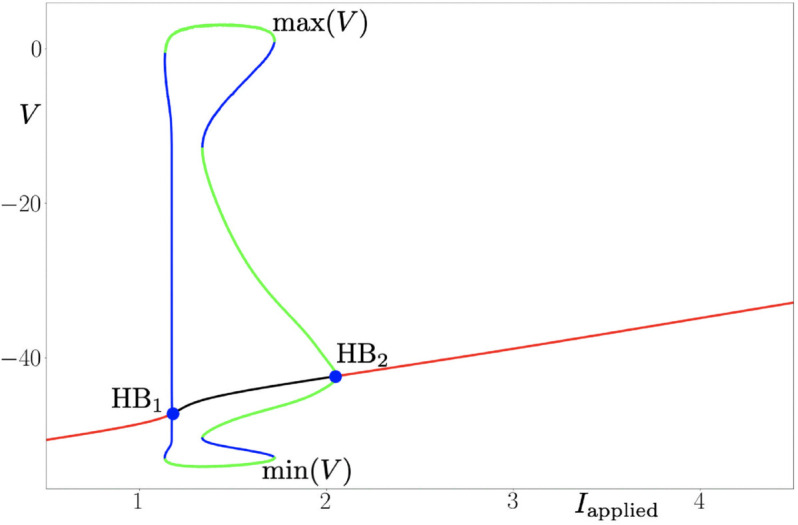
Bifurcation diagram of the system with 3 states, and with respect to parameter $I_{\text{applied}}\equiv u$. Red (resp. black) segments of the curve of equilibria denote stable (resp. unstable) branches. As the applied current $I_{\text{\{applied}}\}$ is increased, oscillations (spikes) appear through a subcritical Hopf (HB_1_) and then disappear through a supercritical Hopf bifurcation (HB_2_). Parameter values are: $\tau_{min}^{1,3}=0.33$ ms, $\tau_{min}^{3,1}=0.33$ ms, $\tau_{min}^{2,3}=1.0$ ms, $V_{\text{half}}^{1,3}=-51\text{mV}$, $V_{\text{half}}^{3,1}=-42\text{mV}$,$V_{\text{half}}^{1,3}=-53\text{mV}$, *k*^1,3^ = −2,0 mV, *k*^3,1^ = 1,0 mV, *k*^2,3^ = −1,0 mV, $\tau_{max}^{2,3}=100$ ms, $V_{Na}=65$ mV, $V_{K}=-70$ mV, $V_{L}=-64.96$ mV, ${\bar{g}}_{Na}=2.28\mu$S/cm^2^, ${\bar{g}}_{DR}=0.76\mu$S/cm^2^, ${\bar{g}}_{A}=8.36\mu$S/cm^2^, ${\bar{g}}_{L}=0.048\mu$S/cm^2^, *s* = 0.2 cm^2^, $V_{us}=-49$ mV, C=0.7μF/cm^2^, *A*^1,2^ = 3 ms^−1^. Initial conditions are : $V_{0}=-65$, *x*_1_ = 0, *x*_2_ = 0, *n*_0_ = 0.00128, *y*_*K*,0_ = 0.47, *n*_*A*,0_ = 0.079, *l*_*A*,0_ = 0.85.

With the model introduced above, it is possible to reproduce the phenomenon of “flipping”, and its appearance is linked to the number of states for the sodium channel of the model. With three states for this channel, the model generates spikes in narrower range of the applied current than with four states, however, the action potentials are almost the same. The model displays one periodic regime upon variations of the applied current, and this regime is bounded in parameter space by two Hopf bifurcations (one subcritical and one supercritical, respectively); see Fig 8.

Namely, a family of low-voltage equilibria (rest states of the neuron) destabilizes and gives way to a family of initially unstable limit cycles via a subcritical Hopf bifurcation (HB_1_) which occurs at an input current value $I_{\text{applied}}\approx 1.2$, before these cycles become stable (spiking states of the neuron) through a fold-of-cycle bifurcation (unlabeled). At a higher value of the input current, the branch of stable cycles disappears via a supercritical Hopf bifurcation (HB_2_) at $I_{\text{applied}}\approx 2$. This scenario is compatible with type-2 neural excitability as observed, e.g., in the classical Hodgkin-Huxley model.

**Fig 9:**
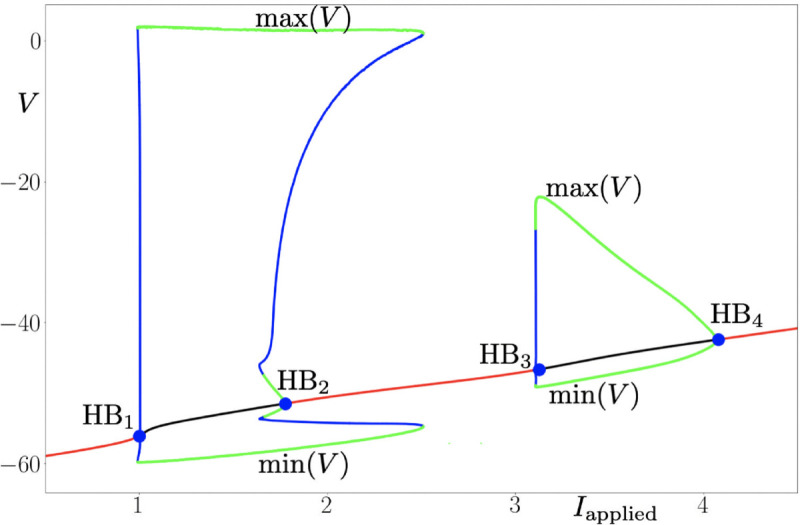
Bifurcation diagram of the system with 4 states, and with respect to parameter $I_{\text{applied}}\equiv u$. Red (resp. black) segments of the curve of equilibria denote stable (resp. unstable) branches. As the applied current $I_{\text{\{applied}}\}$ is increased, oscillations (spikes) appear through a subcritical Hopf (HB_1_) and then disappear through a supercritical Hopf bifurcation (HB_2_). Then, the scenario appears for higher values of $I_{\text{\{applied}}\}$: oscillations appear through a subcritical Hopf (HB_3_) and then disappear through a supercritical Hopf (HB_4_) Parameter values are: $\tau_{min}^{1,3}=0.33$ ms, $\tau_{min}^{3,1}=0.33$ ms, $\tau_{min}^{2,3}=1$ ms, $\tau_{min}^{1,4}=0.33$ ms, $\tau_{min}^{3,4}=1$ ms, $\tau_{min}^{4,1}=0.33$ ms, $V_{\text{half}}^{1,3}=-51\text{mV}$, $V_{\text{half}}^{3,1}=-57\text{mV}$,$V_{\text{half}}^{1,3}=-53\text{mV}$, $V_{\text{half}}^{1,4}=-57\text{mV}$, $V_{\text{half}}^{3,4}=-60\text{mV}$, $V_{\text{half}}^{4,1}=-51\text{mV}$, *k*^1,3^ = −2 mV, *k*^3,1^ = 1 mV, *k*^2,3^ = −1 mV, *k*^1,4^ = −2 mV, *k*^3,4^ = −1 mV, *k*^4,1^ = 1,0 mV, $\tau_{max}^{2,3}=100$ ms, $\tau_{max}^{3,4}=100$ ms, $V_{Na}=65$ mV, $V_{K}=-70$ mV, $V_{L}=-64.96$ mV, ${\bar{g}}_{Na}=2.28\mu$S/cm^2^, ${\bar{g}}_{DR}=0.76\mu$S/cm^2^, ${\bar{g}}_{A}=8.36\mu$S/cm^2^, ${\bar{g}}_{L}=0.048\mu$S/cm^2^, *s* = 0.2 cm^2^, $V_{us}=-49$ mV, C=0.7μF/cm^2^, *A*^1,2^ = 3 ms^−1^. Initial conditions are : $V_{0}=-65$, *x*_1_ = 0, *x*_2_ = 0, *n*_0_ = 0.00128, *y*_*K*,0_ = 0.47, *n*_*A*,0_ = 0.079, *l*_*A*,0_ = 0.85.

In contrast, when the number of states is increased to four, the two Hopf bifurcations from the previous scenario are still present, for similar values of applied current (HB_1_, HB_2_), however a second periodic regime appears, bounded in parameter space by a second pair of Hopf bifurcations, at higher values of $I_{\text{applied}}$; see Fig 9. Indeed, at an input current value $I_{\text{applied}}\approx 3.2$, the stable solution destabilizes again and gives way to a family of stable limit cycles via a supercritical Hopf (HB_3_). This branch of stable cycles disappears via a subcritical Hopf bifurcation (HB_4_) at $I_{\text{applied}}\approx 4.1$. The two pairs of Hopf bifurcations are separated by a regime where the model admits a stable stationary state with voltage higher than the firing threshold, hence a DB state. Therefore, this second periodic regime arising at larger values of the input current is therefore compatible with the “flipping” phenomenon reported in the present work. The 3-state and 4-state Na^ + ^ channel models also differ in their apparent activation and inactivation properties. Indeed, the 4-state model presents circa 5 mV hyperpolarizing shifts in both activation and inactivation curves compared to the 3-state model, together with a larger overlap (window current). These differences may also contribute to the phenomenon of “flipping" (see S2A, S2B Fig).

## Discussion

Electrophysiological criteria, such as intrinsic membrane properties, excitability class represented by firing patterns and transition to depolarization block are very useful tools to identify and to distinguish different types of neurons that are sharing anatomical and functional similarities. Therefore, the primary goal of this study was to explore firing characteristics and entry into DB of granule cells, as these neurons exhibit clear electrophysiological differences due to their location within the dentate gyrus and their stage of maturation (described in the Introduction).

Depolarization block of granule cells is very rarely discussed in experimental findings probably because the range of input currents in which GCs transition to DB could be considered unphysiological, especially in the case of mature GCs. However, a model developed for hippocampal CA1 region by Bianchi suggests that even background synaptic activity in the gamma range involving less than 3 percent of the total number of excitatory synaptic inputs converging on any given CA1 pyramidal neuron, could easily generate an aggregate peak input current larger than 1 nA [4]. This finding makes the DB a very important feature of any neuron, because it is highly probable that in large active networks, such as in the hippocampus, numerous cells will likely be in a DB at any time, therefore their silent state could influence the activity of the entire network. Thus, we believe that DB in granule cells is of great importance and contributes to the dentate gyrus’ filtering abilities.

Many studies focusing on the excitability of granule cells reported two main types of firing patterns that GCs generate in response to increasing current injections. Typically, type-1 DG granule cells are exhibiting spikes starting from a moderate intensity of stimulation and increasing in frequency following step increments. At current injections of high intensity, these cells are lacking sustained firing, action potentials become progressively smaller in amplitude and GCs are entering DB at a relatively early stage (200-250 pA). In contrast, type-2 DG granule cells are usually described as firing throughout all the current steps without failure (not entering DB) and exhibiting firing frequencies that are linearly proportional to the intensity of the current step, i.e. the maximum number of action potentials is always observed for the highest current intensity [27,28,34]. Based on the results presented here, we observed that neurons assigned to LFC group resemble the type-1 cells reported by others and neurons from HFC group could be classified as type 2. In our experimental protocol, however, we used a wider range of current intensities, therefore we were able to observe the transition of granule cells belonging to HFC group into DB at a later stage (450-500 pA). This approach of applying current intensities greater than 300 pA, is not typically used by others, therefore only linear firing patterns of type-2 GCs are most commonly described in other experimental findings.

Under physiological conditions, neurons fire in response to the activation of synaptic conductances. In electrophysiological experiments, neuronal characteristics are usually probed in current-clamp conditions, which cannot fully reflect the synaptic activation because the injected current cannot mimic changes of membrane conductance, if only it is not voltage-dependent. The conductance change provides shunting effect, whose importance is shown, for instance, in visual cortex studies [35,36]. Such characteristics of neuronal activity as the firing rate and the spike shape parameters are more fully expressed by their dependence on both signals, the synaptic current and synaptic conductance, or, the injected voltage-independent current and conductance [37,38]. These signals determine the first two, voltage-independent and linearly voltage-dependent components of the total current received by a neuron from synaptic input [37]. Therefore, in our study we used the dynamic-clamp technique, because it can provide both input signals, the synaptic current and the synaptic conductance [33,39]. The dynamic-clamp stimulation protocols used in our study were based on our previous work [40,41] and the work of others [33,42,43].

Every neuron has sodium and potassium currents and the interaction between these currents results in different transitions between spiking and the silent state. This is why, in the present work, we studied the effects of additional Na- and KM-channels on firing rates and DB of GCs. In the case of rather “weak" LFC with a low maximum firing rates and narrow domain of spiking, the additional Na- and KM-channels increased the firing rate and enlarged the domain so that it resembled the firing pattern of HFC. Interestingly, in the case of HFC, adding Na/KM channels had no effect on maximum firing rate with the exception of one neuron. Moreover, the addition of extra Na/KM channels had no influence on the initial transition to depolarization block, except for “flipping” cells. In the LFC group we found a significant increase in the AP amplitude and that change was driven only by the increase in the peak of the action potential, as the resting membrane potential remained unaffected. The addition of Na/KM channels also did not affect the input resistance.

The discovery of the “flipping” phenomenon is the major finding of this study. As mentioned in the Results section, we defined “flipping” as the ability of certain neurons to overcome the initial depolarization block in order to start generating trains of spikes at larger injected current steps before finally reaching another depolarization block. To the best of our knowledge, this neuronal behavior has not been previously reported in other experimental studies.

Importantly, we were able to reproduce the “flipping” phenomenon in our computational model, reaching the conclusion that the appearance of “flipping” is linked to the number of states for the sodium channel of the model. The voltage-dependent sodium current was approximated by the 4-state Markov model from [30], and its reduction to the 3-state version. The 4-state model describes two closed, one open and one inactivated states of the channel. This version of the model shows a sharp threshold over a 5 to 10 mV range, depending on the recent history of the channel, and the ability to recover from inactivation during repolarizations positive to earlier thresholds, without a large window current, therefore the available threshold depends on the history of firing, shifting down during the course of spike repolarization without giving a strong window current.

With three states for the Na channel, the model displays one periodic regime upon variations of the applied current, and this regime is bounded in parameter space by Hopf bifurcations. This scenario describes a typical non-flipping cell. However, when the number of states is increased to four, a second periodic regime appears, bounded in parameter space by a second pair of Hopf bifurcations occurring at higher values of the applied current. The two pairs of Hopf bifurcations are separated by a regime where the model admits a stable stationary state resembling an initial depolarization block. The presence of the second periodic regime arising at larger values of the input current is compatible with the “flipping” phenomenon, therefore we believe that the appearance of “flipping” is due to the number of sodium channel states. The 4-state channel model is the minimal one that provides multiple (two) closed states. Since we explain the “flipping" effect by such multiple states of sodium channels, we do not expect this effect in any of the one-closed-state models, including the classical Hodgkin-Huxley model. At the same time, presuming that the natural sodium channels also have multiple states, they can expose natural “flipping". In fact, we have observed perturbations in firing patterns of several neurons while recording in control conditions (no extra channels) that could indicate “flipping" (visible in Fig 6D and 6E), however these “flipping"-like perturbations were present when the neuron was not in a full depolarization block. Because we defined “flipping" as the ability to overcome the full DB, where the cell generates only one action potential in a response to a current step and then a train of APs in response to the next step, the presence of “flipping"-like behavior in control conditions was not clear enough for us to classify and describe that observation as natural “flipping". Therefore, we conclude that in the present work we have not observed what we can call natural “flippers", as the clear “flipping" phenomenon was present when the neurons were recorded only under the conditions of additional channels. However, this observation does not exclude the possibility of the existence of neurons able to express the “flipping" behavior without extra Na and KM channels.

Heterogeneities in intrinsic excitability and firing patterns of granule cells have been frequently reported in the context of neurogenesis. As we described in the Introduction section, the intrinsic firing properties and ionic conductances in GCs are thought to reflect their developmental stage and maturation. Immature neurons do not have a well-defined excitability identity (i.e. fixed conductivities) and rather have a fluid conductivity (akin to properties of stem cells), which enables them to explore the landscape of excitability types (1, 2 or 3). The final stage of maturation is stabilized via effectively being programmed by the environment set by local neuronal circuits in the DG. There is a general agreement that GCs expressing less mature phenotype, previously described by others as type-1 cells, are reaching a maximum number of spikes with current steps of moderate intensity, therefore entering depolarization block rather early. On the other hand, GCs classified as type-2 are characterized by a linear firing in response to increasing current injections [27,28]. It has been also recently reported that GCs located in different DG subregions exhibit different firing patterns [44]. Dentate gyrus, within each location along its dorso-ventral span, is anatomically segregated into three different sectors: the suprapyramidal blade, the crest region, and the infrapyramidal blade [45]. Across these sectors, granule cells manifest considerable heterogeneities in their intrinsic excitability, temporal summation, action potential characteristics, and frequency-dependent response properties. Having this in mind, the majority of our recordings were performed in the dorsal blade of the dentate gyrus within the crest. Therefore, we believe that the presence of two subpopulations of GCs described here (LFC vs HFC) is rather due to a different stage of the maturation process of recorded cells than a different location within the DG. This is also supported by the differences we observed in input resistance between LFC and HFC groups (see Introduction). Moreover, we believe that the observed “flipping” phenomenon could be correlated with neuronal maturation, especially when vast majority of “flipping” cells were belonging to LFC group resembling type-1 cells described by others and characterized as having an immature phenotype. However, without a proper “birth dating" technique (for example by using retroviral injections), which was not implemented here, we cannot draw a firm conclusion on this point.

We conjecture that the “flipping” behavior observed here is induced by external electrical input (or in general electrochemical environment) that effectively pushes neurons to jump between classes of excitability. More research in the future will be required in order to describe the “flipping” phenomenon in greater details and answer outstanding open questions: What is the possible role of “flipping”? Is “flipping” correlated with neuronal maturation? And finally, can this phenomenon be observed in other types of neurons in different brain regions?

## Supporting information

S1 Fig (TIFF)

S2 Fig (TIFF)

S1 Dataset (CSV)

## References

[1] McCormick DA, Contreras D. On the cellular and network bases of epileptic seizures. Annu Rev Physiol. 2001;63:815–46. doi: 10.1146/annurev.physiol.63.1.815 11181977

[2] Dichter MA, Ayala GF. Cellular mechanisms of epilepsy: a status report. Science. 1987;237(4811):157–64. doi: 10.1126/science.3037700 3037700

[3] Grace AA, Bunney BS, Moore H, Todd CL. Dopamine-cell depolarization block as a model for the therapeutic actions of antipsychotic drugs. Trends Neurosci. 1997;20(1):31–7. doi: 10.1016/S0166-2236(96)10064-3 9004417

[4] Bianchi D, Marasco A, Limongiello A, Marchetti C, Marie H, Tirozzi B, et al. On the mechanisms underlying the depolarization block in the spiking dynamics of CA1 pyramidal neurons. J Comput Neurosci. 2012;33(2):207–25. doi: 10.1007/s10827-012-0383-y 22310969

[5] Kuznetsova AY, Huertas MA, Kuznetsov AS, Paladini CA, Canavier CC. Regulation of firing frequency in a computational model of a midbrain dopaminergic neuron. J Comput Neurosci. 2010;28(3):389–403. doi: 10.1007/s10827-010-0222-y 20217204 PMC2929809

[6] Tucker KR, Huertas MA, Horn JP, Canavier CC, Levitan ES. Pacemaker rate and depolarization block in nigral dopamine neurons: a somatic sodium channel balancing act. J Neurosci. 2012;32(42):14519–31. doi: 10.1523/JNEUROSCI.1251-12.2012 23077037 PMC3494994

[7] Qian K, Yu N, Tucker KR, Levitan ES, Canavier CC. Mathematical analysis of depolarization block mediated by slow inactivation of fast sodium channels in midbrain dopamine neurons. J Neurophysiol. 2014;112(11):2779–90. doi: 10.1152/jn.00578.2014 25185810 PMC4254877

[8] Economo MN, Fernandez FR, White JA. Dynamic clamp: alteration of response properties and creation of virtual realities in neurophysiology. J Neurosci. 2010;30(7):2407–13. doi: 10.1523/JNEUROSCI.5954-09.2010 20164323 PMC2837935

[9] Espósito MS, Piatti VC, Laplagne DA, Morgenstern NA, Ferrari CC, Pitossi FJ, et al. Neuronal differentiation in the adult hippocampus recapitulates embryonic development. J Neurosci. 2005;25(44):10074–86. doi: 10.1523/JNEUROSCI.3114-05.2005 16267214 PMC6725804

[10] Ge S, Goh ELK, Sailor KA, Kitabatake Y, Ming G, Song H. GABA regulates synaptic integration of newly generated neurons in the adult brain. Nature. 2006;439(7076):589–93. doi: 10.1038/nature04404 16341203 PMC1420640

[11] Toni N, Laplagne DA, Zhao C, Lombardi G, Ribak CE, Gage FH, et al. Neurons born in the adult dentate gyrus form functional synapses with target cells. Nat Neurosci. 2008;11(8):901–7. doi: 10.1038/nn.2156 18622400 PMC2572641

[12] Dieni CV, Nietz AK, Panichi R, Wadiche JI, Overstreet-Wadiche L. Distinct determinants of sparse activation during granule cell maturation. J Neurosci. 2013;33(49):19131–42. doi: 10.1523/JNEUROSCI.2289-13.2013 24305810 PMC3850038

[13] Overstreet Wadiche L, Bromberg DA, Bensen AL, Westbrook GL. GABAergic signaling to newborn neurons in dentate gyrus. J Neurophysiol. 2005;94(6):4528–32. doi: 10.1152/jn.00633.2005 16033936

[14] Tozuka Y, Fukuda S, Namba T, Seki T, Hisatsune T. GABAergic excitation promotes neuronal differentiation in adult hippocampal progenitor cells. Neuron. 2005;47(6):803–15. doi: 10.1016/j.neuron.2005.08.023 16157276

[15] Bielefeld P, Durá I, Danielewicz J, Lucassen PJ, Baekelandt V, Abrous DN, et al. Insult-induced aberrant hippocampal neurogenesis: functional consequences and possible therapeutic strategies. Behav Brain Res. 2019;372:112032. doi: 10.1016/j.bbr.2019.112032 31199935

[16] Hüttmann K, Sadgrove M, Wallraff A, Hinterkeuser S, Kirchhoff F, Steinhäuser C, et al. Seizures preferentially stimulate proliferation of radial glia-like astrocytes in the adult dentate gyrus: functional and immunocytochemical analysis. Eur J Neurosci. 2003;18(10):2769–78. doi: 10.1111/j.1460-9568.2003.03002.x 14656326

[17] Indulekha CL, Sanalkumar R, Thekkuveettil A, James J. Seizure induces activation of multiple subtypes of neural progenitors and growth factors in hippocampus with neuronal maturation confined to dentate gyrus. Biochem Biophys Res Commun. 2010;393(4):864–71. doi: 10.1016/j.bbrc.2010.02.101 20171185

[18] Schmidt-Hieber C, Jonas P, Bischofberger J. Enhanced synaptic plasticity in newly generated granule cells of the adult hippocampus. Nature. 2004;429(6988):184–7. doi: 10.1038/nature02553 15107864

[19] Ge S, Pradhan DA, Ming G-L, Song H. GABA sets the tempo for activity-dependent adult neurogenesis. Trends Neurosci. 2007;30(1):1–8. doi: 10.1016/j.tins.2006.11.001 17116335

[20] Aimone JB, Deng W, Gage FH. Resolving new memories: a critical look at the dentate gyrus, adult neurogenesis, and pattern separation. Neuron. 2011;70(4):589–96. doi: 10.1016/j.neuron.2011.05.010 21609818 PMC3240575

[21] Marín-Burgin A, Mongiat LA, Pardi MB, Schinder AF. Unique processing during a period of high excitation/inhibition balance in adult-born neurons. Science. 2012;335(6073):1238–42. doi: 10.1126/science.1214956 22282476 PMC3385415

[22] Brunner J, Neubrandt M, Van-Weert S, Andrási T, Kleine Borgmann FB, Jessberger S, et al. Adult-born granule cells mature through two functionally distinct states. Elife. 2014;3:e03104. doi: 10.7554/eLife.03104 25061223 PMC4131194

[23] Dieni CV, Panichi R, Aimone JB, Kuo CT, Wadiche JI, Overstreet-Wadiche L. Low excitatory innervation balances high intrinsic excitability of immature dentate neurons. Nat Commun. 2016;7:11313. doi: 10.1038/ncomms11313 27095423 PMC4843000

[24] Mongiat LA, Espósito MS, Lombardi G, Schinder AF. Reliable activation of immature neurons in the adult hippocampus. PLoS One. 2009;4(4):e5320. doi: 10.1371/journal.pone.0005320 19399173 PMC2670498

[25] van Praag H, Schinder AF, Christie BR, Toni N, Palmer TD, Gage FH. Functional neurogenesis in the adult hippocampus. Nature. 2002;415(6875):1030–4. doi: 10.1038/4151030a 11875571 PMC9284568

[26] Parent JM, Yu TW, Leibowitz RT, Geschwind DH, Sloviter RS, Lowenstein DH. Dentate granule cell neurogenesis is increased by seizures and contributes to aberrant network reorganization in the adult rat hippocampus. J Neurosci. 1997;17(10):3727–38. doi: 10.1523/JNEUROSCI.17-10-03727.1997 9133393 PMC6573703

[27] Ambrogini P, Lattanzi D, Ciuffoli S, Agostini D, Bertini L, Stocchi V, et al. Morpho-functional characterization of neuronal cells at different stages of maturation in granule cell layer of adult rat dentate gyrus. Brain Res. 2004;1017(1–2):21–31. doi: 10.1016/j.brainres.2004.05.039 15261095

[28] Nenov MN, Laezza F, Haidacher SJ, Zhao Y, Sadygov RG, Starkey JM, et al. Cognitive enhancing treatment with a PPARγ agonist normalizes dentate granule cell presynaptic function in Tg2576 APP mice. J Neurosci. 2014;34(3):1028–36. doi: 10.1523/JNEUROSCI.3413-13.2014 24431460 PMC3891946

[29] Desroches M, Rinzel J, Rodrigues S. Classification of bursting patterns: a tale of two ducks. PLoS Comput Biol. 2022;18(2):e1009752. doi: 10.1371/journal.pcbi.1009752 35202391 PMC8870467

[30] Borg-Graham LJ. In: Ulinski PS, Jones EG, Peters A, editors. Interpretations of Data and Mechanisms for Hippocampal Pyramidal Cell Models. Boston, MA: Springer US; 1999. p. 19–138. 10.1007/978-1-4615-4903-1_2

[31] Chizhov AV, Graham LJ. Population model of hippocampal pyramidal neurons, linking a refractory density approach to conductance-based neurons. Phys Rev E Stat Nonlin Soft Matter Phys. 2007;75(1 Pt 1):011924. doi: 10.1103/PhysRevE.75.011924 17358201

[32] Milescu LS, Yamanishi T, Ptak K, Mogri MZ, Smith JC. Real-time kinetic modeling of voltage-gated ion channels using dynamic clamp. Biophys J. 2008;95(1):66–87. doi: 10.1529/biophysj.107.118190 18375511 PMC2426646

[33] Graham LJ, Schramm A. In vivo dynamic-clamp manipulation of extrinsic and intrinsic conductances: functional roles of shunting inhibition and IBK in rat and cat cortex. In: Bal T, Destexhe A, editors. Dynamic-clamp: from principles to applications. New York: Springer; 2009.

[34] Jiang N, Cupolillo D, Grosjean N, Muller E, Deforges S, Mulle C, et al. Impaired plasticity of intrinsic excitability in the dentate gyrus alters spike transfer in a mouse model of Alzheimer’s disease. Neurobiol Dis. 2021;154:105345. doi: 10.1016/j.nbd.2021.105345 33766653

[35] Monier C, Chavane F, Baudot P, Graham LJ, Frégnac Y. Orientation and direction selectivity of synaptic inputs in visual cortical neurons: a diversity of combinations produces spike tuning. Neuron. 2003;37(4):663–80. doi: 10.1016/s0896-6273(03)00064-3 12597863

[36] Chizhov AV, Graham LJ. A strategy for mapping biophysical to abstract neuronal network models applied to primary visual cortex. PLoS Comput Biol. 2021;17(8):e1009007. doi: 10.1371/journal.pcbi.1009007 34398895 PMC8389851

[37] Pokrovskii AN. Effect of synapse conductivity on spike development. Biofizika. 1978;23(4):649–53. 209831

[38] Shriki O, Hansel D, Sompolinsky H. Rate models for conductance-based cortical neuronal networks. Neural Comput. 2003;15(8):1809–41. doi: 10.1162/08997660360675053 14511514

[39] Destexhe A, Bal T. Dynamic-clamp: From principles to applications. New York: Springer; 2009.

[40] Chizhov AV, Malinina E, Druzin M, Graham LJ, Johansson S. Firing clamp: a novel method for single-trial estimation of excitatory and inhibitory synaptic neuronal conductances. Front Cell Neurosci. 2014;8:86. doi: 10.3389/fncel.2014.00086 24734000 PMC3973923

[41] Smirnova EY, Zaitsev AV, Kim KK, Chizhov AV. The domain of neuronal firing on a plane of input current and conductance. J Comput Neurosci. 2015;39(2):217–33. doi: 10.1007/s10827-015-0573-5 26278407

[42] Fernandez FR, White JA. Reduction of spike afterdepolarization by increased leak conductance alters interspike interval variability. J Neurosci. 2009;29(4):973–86. doi: 10.1523/JNEUROSCI.4195-08.2009 19176806 PMC2723775

[43] Fernandez FR, White JA. Gain control in CA1 pyramidal cells using changes in somatic conductance. J Neurosci. 2010;30(1):230–41. doi: 10.1523/JNEUROSCI.3995-09.2010 20053905 PMC2865889

[44] Mishra P, Narayanan R. Heterogeneities in intrinsic excitability and frequency-dependent response properties of granule cells across the blades of the rat dentate gyrus. J Neurophysiol. 2020;123(2):755–72. doi: 10.1152/jn.00443.2019 31913748 PMC7052640

[45] Amaral DG, Scharfman HE, Lavenex P. The dentate gyrus: fundamental neuroanatomical organization (dentate gyrus for dummies). Prog Brain Res. 2007;163:3–22. doi: 10.1016/S0079-6123(07)63001-5 17765709 PMC2492885

